# Supplementary figures and images for: In silico analysis on the functional and structural impact of Rad50 mutations involved in DNA strand break repair
Source: PeerJ. 2020 May 22;8:e9197. doi: 10.7717/peerj.9197 (PMC7247530; doi:10.7717/peerj.9197)

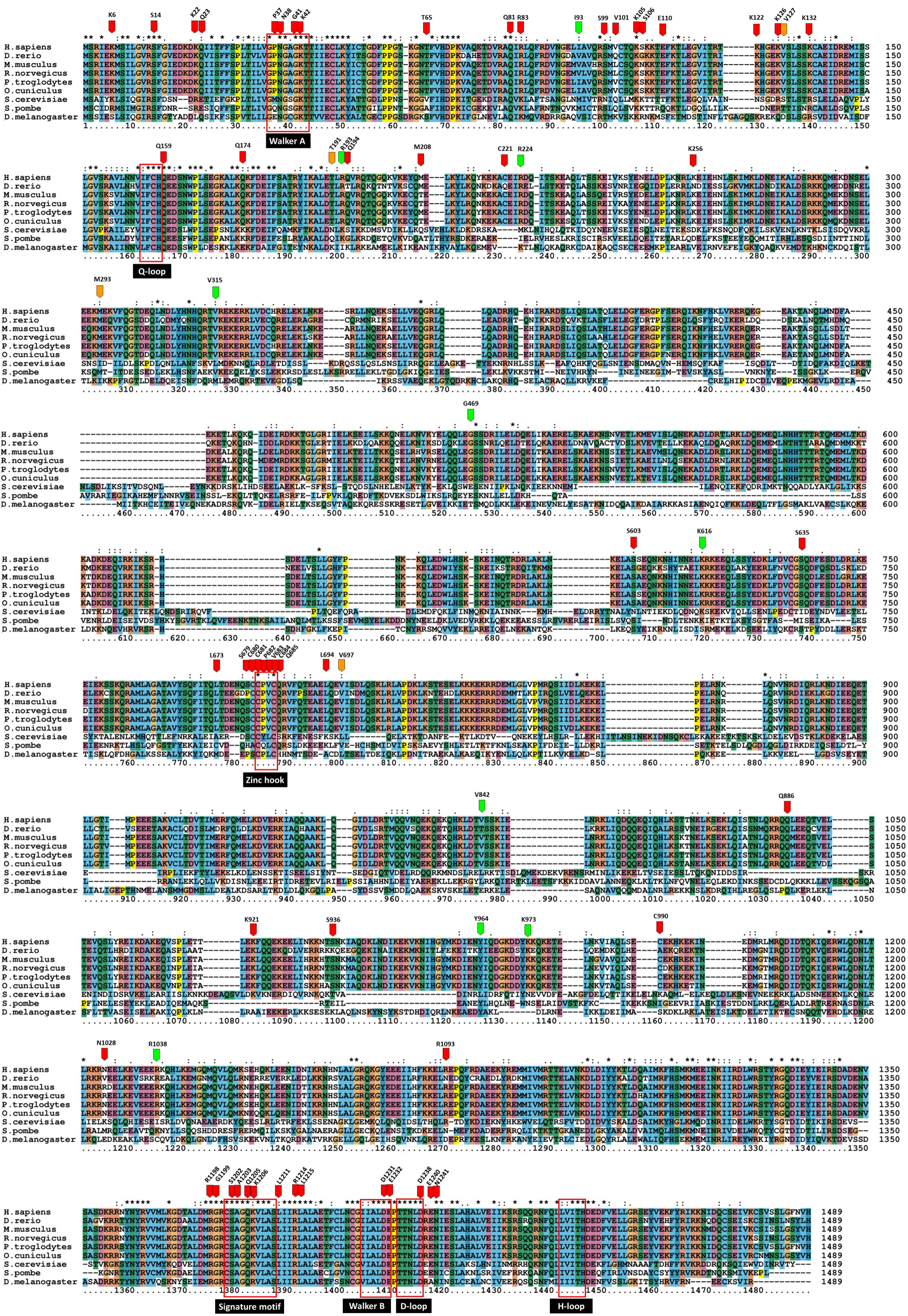

Supplement: Supplemental Information 1 — All the residues identified are marked in the multiple sequence alignment. Red arrows indicate mutations that were identified by previous studies whereas green arrows show SNPs identified from SNPeffect 4.0 database. Four orange arrows at residues V127, T191, M293 and V697 represent overlapping mutations identified from both previous research and SNPs database. [file peerj-08-9197-s001.pdf]

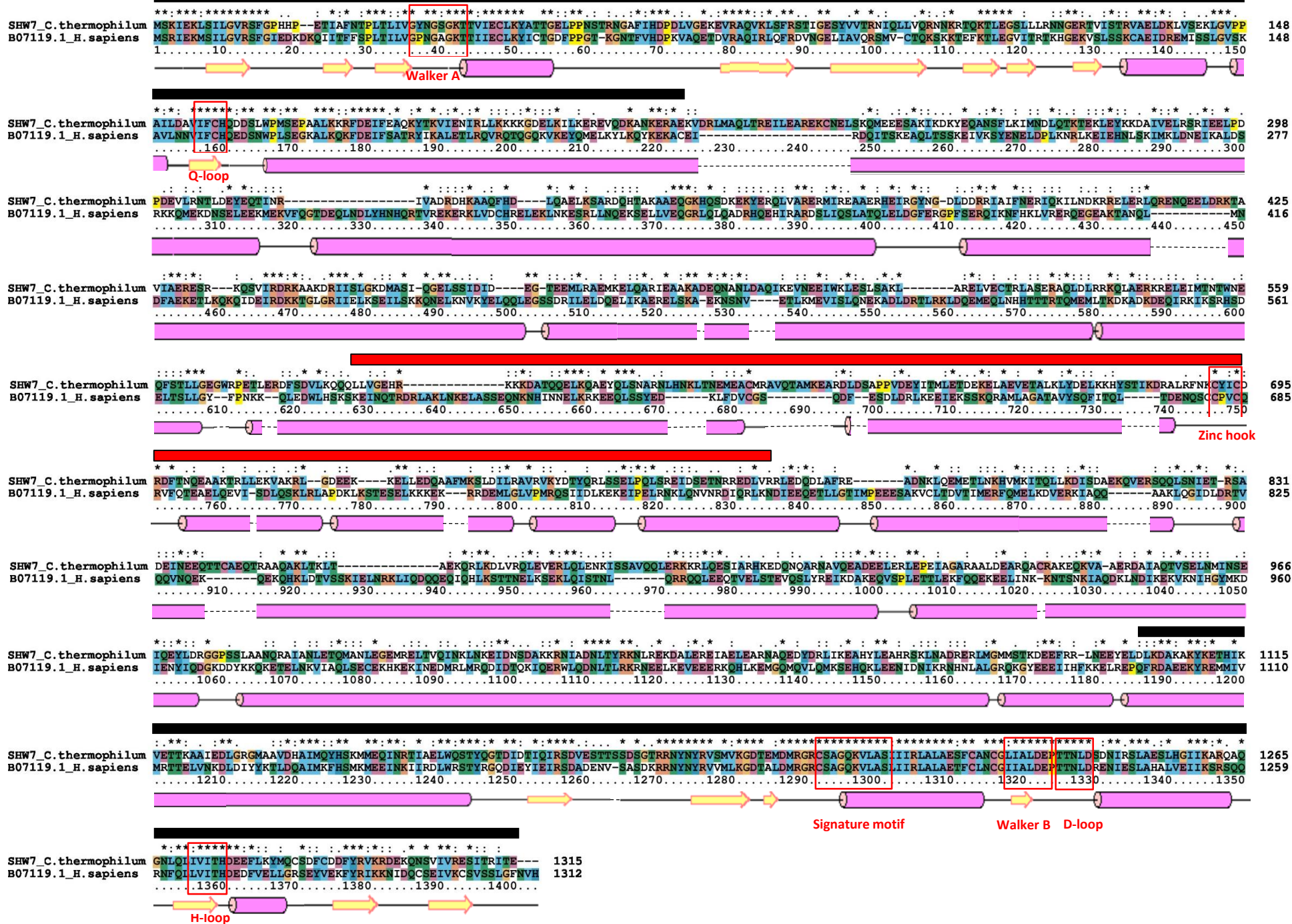

Supplement: Supplemental Information 2 — Alignment data between C. thermophilum and human Rad50 suggests that the amino acid sequences near the six motifs of Rad50 namely Q-loop, Walker B, signature motif, D-loop, Walker A and H-loop (marked with a box) are highly conserved and similar (with 30% sequence identity) (shadowed under a black line), indicating the importance of these motifs to ensure proper function of Rad50. No correlation within the amino acid sequences which conjunct the zinc hook motif (shadowed under a red line) between both species where able to be determined using pairwise alignment. [file peerj-08-9197-s002.pdf]
