# Supplementary material for: In silico analysis on the functional and structural impact of Rad50 mutations involved in DNA strand break repair
Source: PeerJ. 2020 May 22;8:e9197. doi: 10.7717/peerj.9197 (PMC7247530; doi:10.7717/peerj.9197)
Supplement: Supplemental Information 3 — The star indicates known reported mutation in Rad50 that cause deleterious effect. Most residues that were predicted to be neutral are located at the non-conserved positions in the Rad50 protein. [file peerj-08-9197-s003.pdf]

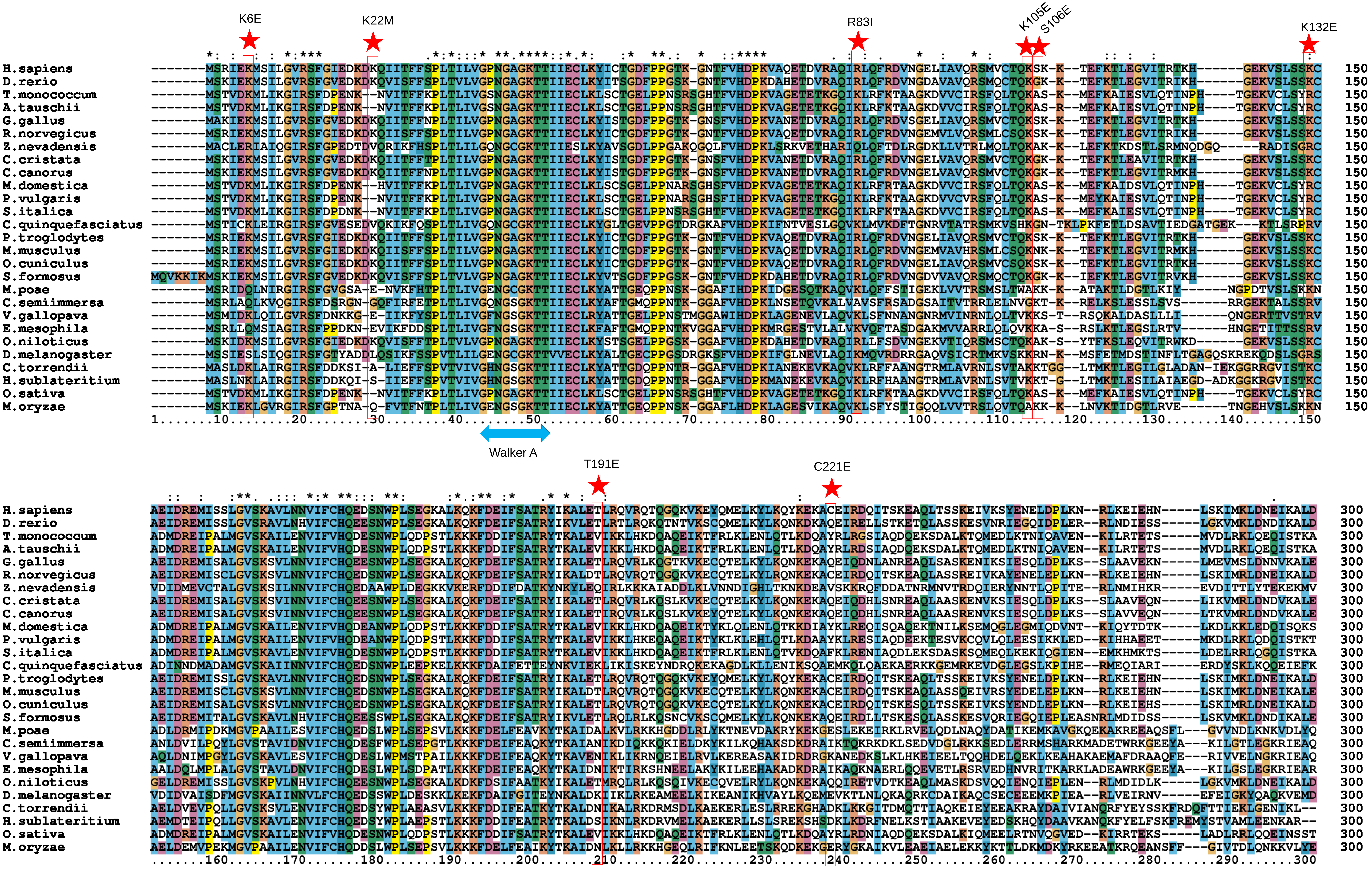
K6E
K22M
R83I
K105E
S106E
K132E
H.sapiens
D.rerio
T.monococcum
A.tauschii
G.gallus
R.norvegicus
Z.nevadensis
C.cristata
C.canorus
M.domestica
P.vulgaris
S.italica
C.quinquefasciatus
P.troglodytes
M.musculus
O.cuniculus
S.formosus
M.poae
C.semiimmersa
V.gallopava
E.mesophila
O.niloticus
D.melanogaster
C.torrendii
H.sublateritium
O.sativa
M.oryzae
Walker A
T191E
C221E

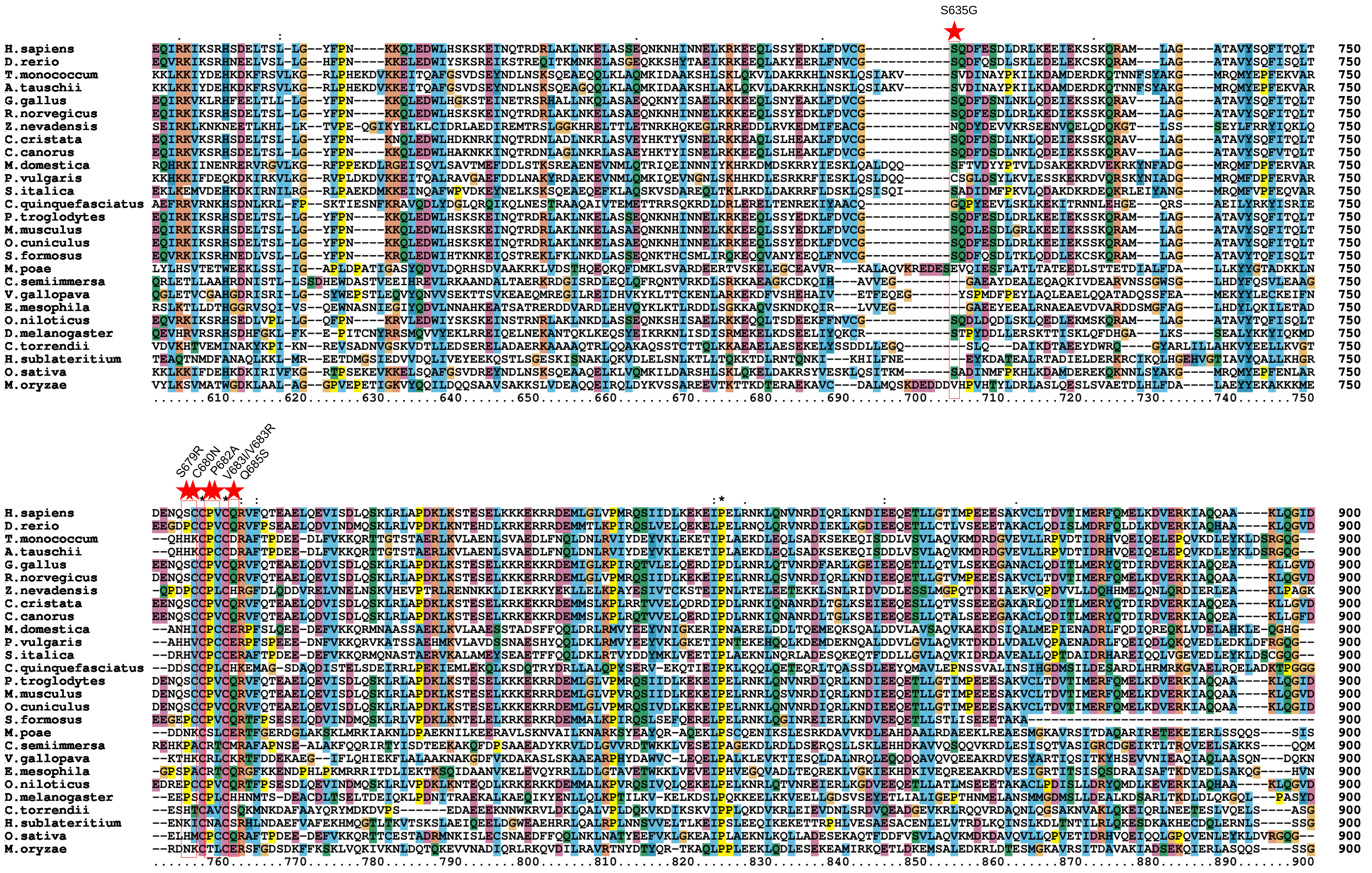
S635G
S679R
C680N
P682A
V683I/V683R
Q685S
H.sapiens
D.rerio
T.monococcum
A.tauschii
G.gallus
R.norvegicus
Z.nevadensis
C.cristata
C.canorus
M.domestica
P.vulgaris
S.italica
C.quinquefasciatus
P.troglodytes
M.musculus
O.cuniculus
S.formosus
M.poae
C.semiimmersa
V.gallopava
E.mesophila
O.niloticus
D.melanogaster
C.torrendii
H.sublateritium
O.sativa
M.oryzae
