# Supplementary material for: In silico analysis on the functional and structural impact of Rad50 mutations involved in DNA strand break repair
Source: PeerJ. 2020 May 22;8:e9197. doi: 10.7717/peerj.9197 (PMC7247530; doi:10.7717/peerj.9197)
Supplement: Supplemental Information 4 — Previous Rad50 mutations have been reported in human, mice, yeast, and bacteria [file peerj-08-9197-s004.docx]

.

| \| Motif \| Mutations \| Organism \| Equivalent to human  residue \| Ref. \| \| --- \| --- \| --- \| --- \| --- \| \| Walker A \| K40A  K40R  K40E \| *S. cerevisiae* \| K42A  K42R  K42E \| [1] \| \| Walker A  (WA)  &  D-loop \| R37A  N38A  D512N  D512A  E514Q  E515A \| T4  bacteriophage \| P37A  N38A  D1238N  D1238A  E1240Q  N1241A \| [2] \| \| ATP binding domain  &  Walker A \| K6E  S14P  R20M  E21K  G39D (WA)  K40E (WA)  V63E  Q79K  K81I  N97D  Q99K  E915V  A930P \| *S. cerevisiae* \| K6E  S14P  K22M  Q23K  G41D  K42E  T65E  Q81K  R83I  S99D  V101K  K921V  S936P \| [3] \| \| ATP binding domain \| K6E =K6E^Sc^  K22M=R20M^Sc^  R83I=K81^Sc^ \| *M. musculus* \| K6E  K22M  R83I \| [4] \| \| Walker A,  Walker B  & Signature motif \| K39R (WA)  K39M (WA)  D303N (WB)  S270R (SM) \| *D. radiodurans* \| K42R  K42M  D1231N  S1202R \| [5] \| \| ATPase binding domain  &  Walker B (WB)  &  Signature motif (SM) \| K115E  K175E  K182E  R94E  K95E  R765E  E798Q (WB)  S768R (SM)  K99E  K108E  K109E  K110E  R125E  K103E  K104E  R131E  R1201E  N190D  S1205E+E1235Q \| *T. maritima*  *S. cerevisiae* \| K132E  T191E  C221E  K105E  S106E  G1199E  E1232Q  S1202R  E110K  E126K  K127E  K122E  K126E  K105E  S106E  K132E  R1198E  T191D  S1202R  +E1232Q \| [6] \| \| Zinc hook \| S679R  P682E  V683R \| *M. musclulus* \| S679R  P682E  V683R \| [7] \| \| Zinc hook \| C680G  C681G  C684G  R686A  C681G+C684G  C680G+C681G+  C684G \| *H. sapiens* \| C680G  C681G  C684G  R686A \| [8] \| \| Zinc hook \| C684N  C685A  P686A  V6871  C688R  Q689S \| *S. cerevisiae* \| C680N  C681A  P682A  V683I  C684R  Q685S \| [9] \| | \| Motif \| Mutations \| Organism \| Equivalent to human  residue \| Ref. \| \| --- \| --- \| --- \| --- \| --- \| \| Zinc hook &  coiled coil domain (ATPase domain) \| C288S  C291S  C312S  K211P  K351P  S183C \| T4  bacteriophage \| C681S  C684S  C990S  K256P  N1028P  M208C \| [10] \| \| Zinc hook  &  coiled coil domain (ATPase domain) \| S685R  Y688E  Y688R  L689R  I680V  K700Q  L703F  V285A  N607Y  N873I  S193F \| *S cerevisiae* \| S679R  P682E  P682R  V683R  L673V  L694Q  V697F  M293A  S603Y  Q886I  Q194F \| [11] \| \| Zink hook \| S635G \| *H. sapiens* \| S635G \| [12] \| \| Signature motif &  ATPase domain \| R797G  R805E  R805W  L802W  L806F  K155A \| *P. furiosus* \| K1206G  R1214E  R1214W  L1211W  L1215F  Q174A \| [13] \| \| Signature motif \| K1187A  K1187E  R1195A  R1195E \| *S.pombe* \| K1206A  K1206E  R1214A  R1214E \| [14] \| \| Signature motif & Q loop \| S793R  Q140H \| *P. furiosus* \| S1202R  Q159H \| [15] \| \| Signature motif \| S471A  S471R  S471M  E472G  E474Q  K475M \| T4  bacteriophage \| S1202A  S1202R  S1202M  A1203G  Q1205E  K1206M \| [16] \| \| Signature motif \| S1205R  S793R  S1202R \| *S. cerevisiae*  *P. furiosus*  *H. sapiens* \| S1202R \| [17] \| \| ATPase domain \| R1093X \| *H. sapiens* \| R1093X \| [18] \| |
| --- | --- | --- | --- | --- | --- | --- | --- | --- | --- | --- | --- | --- | --- | --- | --- | --- | --- | --- | --- | --- | --- | --- | --- | --- | --- | --- | --- | --- | --- | --- | --- | --- | --- | --- | --- | --- | --- | --- | --- | --- | --- | --- | --- | --- | --- | --- | --- | --- | --- | --- | --- | --- | --- | --- | --- | --- | --- | --- | --- | --- | --- | --- | --- | --- | --- | --- | --- | --- | --- | --- | --- | --- | --- | --- | --- | --- | --- | --- | --- | --- | --- | --- | --- | --- | --- | --- | --- | --- | --- | --- | --- | --- | --- | --- | --- | --- | --- | --- | --- | --- | --- |

| References  1. Chen L, Trujillo KM, Van Komen S, Roh DH, Krejci L, Lewis LK, et al. Effect of amino acid substitutions in the rad50 ATP binding domain on DNA double strand break repair in yeast. J Biol Chem. 2005;280: 2620–2627. doi:10.1074/jbc.M410192200  2. De La Rosa Metzere Bierlein, and Scott W. Nelson. An Interaction between the Walker A and D-loop Motifs Is Critical to ATP Hydrolysis and Cooperativity in Bacteriophage. J Biol Chem. 2011;286: 26258–26266. doi:10.1074/jbc.M111.256305  3. Alani E, Padmore R, Kleckner N. Analysis of wild-type and rad50 mutants of yeast suggests an intimate relationship between meiotic chromosome synapsis and recombination. Cell. 1990;61: 419–436. doi:10.1016/0092-8674(90)90524-I  4. Bender CF, Sikes ML, Sullivan R, Huye LE, Le Beau MM, Roth DB, et al. Cancer predisposition and hematopoietic failure in Rad50S/S mice. Genes Dev. 2002;16: 2237–2251. doi:10.1101/gad.1007902  5. Koroleva O, Makharashvili N, Courcelle CT, Courcelle J, Korolev S. Structural conservation of RecF and Rad50: implications for DNA recognition and RecF function. EMBO J. 2007;26: 867–77. doi:10.1038/sj.emboj.7601537  6. Rojowska A, Lammens K, Seifert FU, Direnberger C, Feldmann H. Structure of the Rad 50 DNA double-strand break repair protein in complex with DNA. EMBO J. 2014;33: 2847–2859. doi:10.15252/embj.201488889  7. Roset R, Inagaki A, Hohl M, Brenet F, Lafrance-Vanasse J, Lange J, et al. The Rad50 hook domain regulates DNA damage signaling and tumorigenesis. Genes Dev. 2014;28: 451–462. doi:10.1101/gad.236745.113  8. Cahill D, Carney JP. Dimerization of the Rad50 protein is independent of the conserved hook domain. Mutagenesis. 2007;22: 269–274. doi:10.1093/mutage/gem011  9. He J, Shi LZ, Truong LN, Lu CS, Razavian N, Li Y, et al. Rad50 zinc hook is important for the Mre11 complex to bind chromosomal DNA double-stranded breaks and initiate various DNA damage responses. J Biol Chem. 2012;287: 31747–31756. doi:10.1074/jbc.M112.384750  10. Barfoot T, Herdendorf TJ, Behning BR, Stohr BA, Gao Y, Kreuzer KN, et al. Functional analysis of the bacteriophage T4 Rad50 Homolog (gp46) Coiled-coil Domain. J Biol Chem. 2015;290: 23905–23915. doi:10.1074/jbc.M115.675132  11. Hohl M, Kocha??czyk T, Tous C, Aguilera A, KrEzel A, Petrini JHJ. Interdependence of the Rad50 Hook and Globular domain functions. Mol Cell. 2015;57: 479–492. doi:10.1016/j.molcel.2014.12.018  12. Gatei M, Jakob B, Chen P, Kijas AW, Becherel OJ, Gueven N, et al. ATM protein-dependent phosphorylation of Rad50 protein Regulates DNA repair and cell cycle control. J Biol Chem. 2011;286: 31542–31556. doi:10.1074/jbc.M111.258152  13. Deshpande RA, Williams GJ, Limbo O, Williams RS, Kuhnlein J, Lee JH, et al. ATP-driven Rad50 conformations regulate DNA tethering, end resection, and ATM checkpoint signaling. EMBO J. 2014;33: 482–500. doi:10.1002/embj.201386100  14. Williams GJ, Williams RS, Williams JS, Moncalian G, Arvai AS, Limbo O, et al. ABC ATPase signature helices in Rad50 link nucleotide state to Mre11 interface for DNA repair. Nat Struct Mol Biol. 2011;18: 423–431. doi:10.1038/nsmb0911-1084c  15. Moncalian G, Lengsfeld B, Bhaskara V, Hopfner KP, Karcher A, Alden E, et al. The Rad50 Signature Motif: Essential to ATP Binding and Biological Function. J Mol Biol. 2004;335: 937–951. doi:10.1016/j.jmb.2003.11.026  16. Herdendorf TJ, Nelson SW. Functional evaluation of bacteriophage T4 Rad50 signature motif residues. Biochemistry. 2011;50: 6030–6040. doi:10.1021/bi200184w  17. Bhaskara V, Dupr A, Lengsfeld B, Hopkins BB, Chan A, Lee JH, et al. Rad50 Adenylate Kinase Activity Regulates DNA Tethering by Mre11/Rad50 Complexes. Mol Cell. 2007;25: 647–661. doi:10.1016/j.molcel.2007.01.028  18. Waltes R, Kalb R, Gatei M, Kijas AW, Stumm M, Sobeck A, et al. Human RAD50 Deficiency in a Nijmegen Breakage Syndrome-like Disorder. Am J Hum Genet. 2009;84: 605–616. doi:10.1016/j.ajhg.2009.04.010 |
| --- |
