# Supplementary material for: In silico analysis on the functional and structural impact of Rad50 mutations involved in DNA strand break repair
Source: PeerJ. 2020 May 22;8:e9197. doi: 10.7717/peerj.9197 (PMC7247530; doi:10.7717/peerj.9197)
Supplement: Supplemental Information 5 — Table shows the impact of Rad50 mutation found in known reports from every organism. Each mutation site that equivalent to human residue were determined using pairwise alignment. [file peerj-08-9197-s005.docx]

| Organism &  References | Motif | Mutations | Effects | Equivalent mutation in human residue |
| --- | --- | --- | --- | --- |
| *Saccharomyces cerevisiae* [1] | Walker A | K40A  K40R  K40E | 1. All mutation cause hypersensitivity to DNA damaging agent. Also showed homologous recombinant (HR) defects as Rad50 deletion.   Deficiency in non homologous end joining (NHEJ), defective in ATPase, ATP-dependent, DNA unwinding& ATP stimulated endonuclease activity. | K42A  K42R  K42E |
| T4 bacteriophage [2] | Walker A  (WA)  &  D-loop | R37A  N38A  D512N  D512A  E514Q  E515A | 1. D512H and N38I are the naturally occuring mutation in the Cystic fibrosis transmembrane conductance regulator (CFTR) which lead to cystic fibrosis. 2. Mutation of D512N and D512A (D-loop) and N38A (WA) results in dramatic reduction in ATP affinity, hydrolysis rate and cooperativity. 3. D loop aspartate (D) fx tu stabilize the WA in a position favorable for catalysis. 4. N38 (asparigine) is crucial for mechanism of ATP hydrolysis by increase affinity to ATP & positioning the γ-phosphate of ATP for catalysis.   E514Q, E515A and R37A has little effects on ATP hydrolysis and nuclease activity of Mre11. | P37A  N38A  D1238N  D1238A  E1240Q  N1241A |
| *Saccharomyces cerevisiae* [3] | ATP binding domain  &  Walker A | K6E  S14P  R20M  E21K  G39D (WA)  K40E (WA)  V63E  Q79K  K81I  N97D  Q99K  E915V  A930P | 1. G39D and K40E : Both of this mutation confer a phenotype identical to Rad50 null mutation characterized by total defect in formation of viable spore. 2. G39D and K40E is highly conserved residue and analogous changes eliminate ATP hydrolysis (Personage et al. 1987; Sung et al. 1988 amd Reinstein et al. 1988). 3. From 9 residue located at ATPase at N terminal (K6E, S14P, R20M, E21K, V63E, Q79K, K81I, N97D), only K81I and R20M exhibit total phenotype defects in sporulation but still nearly to WT in term of resistance to MMS (DNA damage reagent). 4. Rad50 null mutation (complete deletion 3.9kb) showed 3 mitotic defects; 1) sensitive to MMS 2) defect in normal vegetative growth even in high rich media. 3) failure in DNA damage that arise spontaneously or during DNA replication.   Double mutation: E915V + A930P showed temperature sensitive. | K6E  S14P  K22M  Q23K  G41D  K42E  T65E  Q81K  R83I  S99D  V101K  K921V  S936P |
| *Mus musculus* [4] | ATP binding domain | K6E =K6E^Sc^  K22M=R20M^Sc^  R83I=K81^Sc^ | 1. R83I and K6E: embryonic lethality. 2. K22M: Mice were viable, but have partial embryonic lethality, growth defect and cancer predisposition. 3. Cell culture derived from mutant mice showed increase spontaneous apoptosis which reduce chromosome stability. This mechanism lead to hematopoietic & spermatogenic depletion in mice.   Shorten lifespan associated with progressive loss of cells; die within 3 month. | K6E  K22M  R83I |
| *Deinococcus radiodurans* [5] | Walker A,  Walker B  & Signature motif | K39R (WA)  K39M (WA)  D303N (WB)  S270R (SM) | 1. K39R=K36R^E.coli^: Mutation prevent ATP hydrolysis but not nucleotide binding. Shown to be critical for RecF function (Sander et al. 1992; Web et al. 1999). 2. K39R=K36M^E.coli^: Mutation prevent ATP binding. 3. D303N: In other SMC proteins, this changes traps ATP in its transition state & stabilized dimerization of the homodimer (Smith et al. 2002).   S270R: Equivalent mutation interfers with ATP dependent dimerization (Moncalian et al. 2004), | K42R  K42M  D1231N  S1202R |
| *Thermotoga maritima* [6]  *Saccharomyces cerevisiae* | ATPase binding domain  &  Walker B (WB)  &  Signature motif (SM) | K115E  K175E  K182E  R94E  K95E  R765E  E798Q (WB)  S768R (SM)  K99E  K108E  K109E  K110E  R125E  K103E  K104E  R131E  R1201E  N190D  S1205E+E1235Q | ***In vitro: Thermotoga maritima***   1. Coiled-coil: K175E, K182E; substantially reduced DNA binding, but K115E almost abolish DNA interaction in vitro. 2. R94E & K95E: Important for DNA binding. 3. R765E: Significantly diminished DNA binding without affecting dimer formation in vitro. 4. E798Q: Showed strong DNA binding in the present of ATP and slightly low affinity to DNA compared to WT in the presence of AMPPNP. 5. S768R: Significantly reduced DNA binding in the presence of AMPPNP.   ***In vivo:*** ***Saccharomyces cerevisiae***   1. S1205R (SM)+E1235Q (WB): unable to rescue the impaired DNA damage response; showed importance function of ATP binding & ATP hydrolysis in the double strand break (DSB) repair activity. 2. K103E, K104E & R131E: Strongly affected DNA binding in vitro but little influence on the DSB repair fx. in vivo. 3. K110E & R125E: Insignificant reduction in plasmic recovery; do not involved in end joning process. 4. K103E, K104E & R131E: Moderate reduction in telomere length. 5. K103E+R131R & R1201E: Significantly reduced telomere length. 6. S1205R: Significantly reduced telomere length. 7. E1235Q: Fully proficient in telomere maintenance.   N190D: No effect on telomere | K132E  T191E  C221E  K105E  S106E  G1199E  E1232Q  S1202R  E110K  E126K  K127E  K122E  K126E  K105E  S106E  K132E  R1198E  T191D  S1202R+E1232Q |
| *Mus musclulus* [7] | Zinc hook | S679R  P682E  V683R | 1. Double mutation S679R and P682E showed reduced zinc affinity and dimerization efficiency. Also showed lethality in mice. Same finding for V683R. Crossbreed with WT showed hydrocephalus, defects in primitive hematopoietic & gametogenic cells.   Therefore, this hook domain strongly influences Mre11 complex dependent DDR signaling, tissue homeostasis and tumorigenesis. | S679R  P682E  V683R |
| *Homo sapiens* [8] | Zinc hook | C680G  C681G  C684G  R686A  C681G+C684G  C680G+C681G+  C684G | 1. 6 mutation has been created (point mutatio, double and triple mutation)   Cystein in hook domain do not required for human Rad50 interaction with itseft and no significant effect on DNA binding. | C680G  C681G  C684G  R686A |
| *Saccharomyces cerevisiae* [9] | Zinc hook | C684N  C685A  P686A  V6871  C688R  Q689S | 1. Mutation 1: C684N + C685A + P686A + V6871 + C688R + Q689S   Mutation 2: C685A + C688A   1. In vitro: mutant unable to bind to double strand break compred to WT.   In vivo: mutant also defective to be recriuted to chromosomal double strand break.   1. Mutant phenotype as severe as Rad50 null mutant.   Rad50 hook mutant severely defects in various DNA damage response includes ATM activation, homologous recombinant, sensitive to irradiation and ATR activation. | C680N  C681A  P682A  V683I  C684R  Q685S |
| T4 bacteriophage [10] | Zinc hook &  coiled coil domain (ATPase domain) | C288S  C291S  C312S  K211P  K351P  S183C | 1. Double mutation of C288S and C291S in T4 is lethal; indicate the ability to bind zinc^2+^ is critical for the functioning of MR complex. 2. Point mutation of zinc hook or coiled coil domain do not affect Mre11 and DNA binding, but their activation of Rad50 ATPase activity is abolish. 3. Deletion of coiled-coil and zinc hook eliminate Mre11 binding & ATPase activation but do not affect basal activity.   Therefore coiled coil enhance ATPase activity, major conduit for communication between Mre11 and Rad50 as well as support nucleoide excision. | C681S  C684S  C990S  K256P  N1028P  M208C |
| *Saccharomyces cerevisiae* [11] | Zinc hook  &  coiled coil domain (ATPase domain) | S685R  Y688E  Y688R  L689R  I680V  K700Q  L703F  V285A  N607Y  N873I  S193F | 1. 3 mutation has been created in zinc hook.   Mutation Rad50^46^: S685R + Y688E; Rad50^48^: S685R + Y688R; Rad50^47^: L689R   1. Sensitivity test: Rad50^46^ and Rad50^47^ shoewd partial sensitivity to MMS, hyroxyurea & camptothesin.   Rad50^48^ showed minimal sensitivity to MMS.   1. Interaction: Rad50^46;^ Rad50-Mre11 interaction was strongly impaired. Rad50^47^ and Rad50^48^ not clearly affected compared to WT. 2. Viabiliy: Sporulation efficiency and viability were severely impaired in Rad50^46^ followed by Rad50^47^. Rad50^48^ close to WT. 3. Rad50^46^ (S685R + Y688E ) also showed partial supression of telomere and meiotic defects.   Mre11 interaction was also partially improved in I680V, K700Q, V285A, N607Y, N873I and S193F. L703F allele restored the interaction, consistent with its pronounced effect on MMS resistance. | S679R  P682E  P682R  V683R  L673V  L694Q  V697F  M293A  S603Y  Q886I  Q194F |
| *Homo sapiens*[12] | Zich hook | S635G | 1. Characterised by chromosomal instability and defective ATM-dependent signalling. 2. The study found that ATM phosphorylates Rad50 at a single site (S635) that plays an important role in signalling for cell cycle control and DNA repair. 3. Even a Rad50 phosphosite-specific mutant (S635G) supported normal activation of ATM in Rad50 deficient cells, but it was defective in correcting DNA damage induced signalling through the ATM-dependent substrate SMC1. 4. This mutant also failed to correct radiosensitivity, DNA double strand break (DSB) repair and an S phase checkpoint defect in Rad50 deficient cells.   Demonstrated that the Rad50 S635 phosphorylation site plays a role in mediating the intra-S checkpoint after irradiation, and it is hypothesised to play a role as an ATM adaptor to regulate downstream ATM signalling. | S635G |
| *Pyrococcus furiosus* [13] | Signature motif &  ATPase domain | R797G  R805E  R805W  L802W  L806F  K155A | 1. L802W: Decrease dimerization in ATP & hydrolysis; also decrease in cleavage site 2. R805E: Increase dimerization and do not have significant proteolysis with WT. 3. In vivo; L802E=I1214W^Sc^ grown well in camptothecin (CPT) and showed rapid DNA repair after induced with HO endonuclease.   R805E=R1217E^Sc^ showed poorly grown in CPT; inability to repair endogenous DNA damage by HR. Also showed defect in resection which stll required 25kb for repair. And showed defect in resection in HO induced.   1. I1214W + R1217E: Robust in promoting NHEJ; same as WT. 2. R1217E improve survival significantly compared WT and I1214W; after generating DSBs across genome. 3. R1217E: separation of function such that the resection are specifically impaired but NHEJ activity are intact& hyperactive; consistent with results in vitro.   I1214W: Nearly WT for all assays. | K1206G  R1214E  R1214W  L1211W  L1215F  Q174A |
| *Schizo-saccharomyces pombe* [14] | Signature motif | K1187A  K1187E  R1195A  R1195E | 1. K1187A: sensitive in higher dose of clastogens. 2. K1187E, R1195A & R1195E: significantly sensitive to clastogen agents and are deleterious as Rad50 null mutation. | K1206A  K1206E  R1214A  R1214E |
| *Pyrococcus furiosus* [15] | Signature motif & Q loop | S793R  Q140H | 1. S793R=S1202R ^H.sapiens^: Analogs to the mutation in CFTR gene that results cystic fibrosis. 2. S793R: This mutation prevent ATP binding & disrupts the communication among the other ATP loops. This structural changes, in turn, altrs th interaction among rad50 monomers and thus prevents Rad50 dimerization. 3. S1202R: In human genes; mutant protein did form MRN complex but significantly dificient in all ATP-dependent enzymatic activities. 4. S1205R ^S. cerevisiae^: Failed to complement Rad50 deletion strain in DNA repair assay in vivo.   Q159H & S1202R: Mutant exhibited normal exonuclease & endonuclease activity, while all of the ATP-dependent activities of the complex were completely abrogated. | S1202R  Q159H |
| T4 bacteriophage [16] | Signature motif | S471A  S471R  S471M  E472G  E474Q  K475M | 1. S471A, S471R &S471M: Reduced the level of DNA activation of ATPase suggesting this residues involved in the allosteric transmission between DNA & ATP binding sites. Decreased ATP cooperativity is vary depend on the side chain alteration; indicated that signature motif involved in communication between ATP site. 2. Same finding for E474Q and K475M.   E472G: No effect on DNA activation. | S1202A  S1202R  S1202M  A1203G  Q1205E  K1206M |
| *Saccharomyces cerevisiae*  *Pyrococcus furiosus*  *Homo sapiens*  [17] | Signature motif | S1205R  S793R  S1202R | 1. Mutation at conserved SM (S1202R) reduced adenylate kinase activity for DNA tethering reaction but proficient in ATP hydrolysis. This mutant resembles Rad50 null strain in term of meiosis and telomere maintenance in S. cerevisiae 2. S793R: Found dificient in ATP-dependent dimer formation & ATP binding (Hofner et al. 2004). Previous finding; low affinity to ATP (Moncalian et al. 2004). 3. S793R: This mutation was modelled after an analogous mutation in CFTR (S549R) that results in cystic fibrosis (Kerem et al. 1989). 4. Compared to P. furiosus; equivalent mutation in S1202R ^H. sapiens^ and S1205 ^S. cerevisiae^ do not inhibit ATPase activity. 5. S1202R & S1205R: Level of adenylate kinase significantly (10-12 fold) lower than WT. 6. S1205R: Its phenotype equivalent to null Rad50 mutation in yeast which respect to MMS & bleomycin resistance (Moncalian et al. 2004). This mutant also fails to complement the sequence in assays of NHEJ in deletion strain in vivo (Zhang & paull 2005). This mutation also do not support spore viability (Bhaskara et al. 2007). Last; the telomere were severely shortened as in Rad50 deletion strain.   S1205R: Mutation showed high level of DNA tethering, but not stimulated by ATP. In contast mutation in Walker A; K40A ^S. cerevisiae^ (K42AHS) completely block all DNA tethering, consistent with prevous report by Chen et al. 2005. | S1202R |
| *Homo sapiens* [18] | ATPase domain | R1093X | 1. Nijmegen Breakage Syndrome like disorder (NBSLD). Event where a patient diagnosed with smaller head (microcephaly), mental retardation, ‘bird like’ face and short stature due to Rad50 dificiency. 2. ToFurther studies of this patient’s fibroblasts and lymphoblastoid cells (LCL) revealed that this patient had RAD50 protein deficiency (Waltes et al., 2009). Sequencing of the RAD50 cDNA of this patient revealed compound heterozygosity for mutations that appeared to have hypomorphic effect.   The mutation (c.3277C/T; p.R1093X) on exon 21 was maternally inherited. This mutation created a premature termination codon, thus producing a truncated RAD50 protein. The second mutation on exon 25 (c.3939A/T) was inherited paternally. This mutation changed the stop codon of RAD50 to a tyrosine codon, thereby producing a larger RAD50 peptide. How these mutations affect the low level of RAD50 protein expression in this patient remains to be elucidated (Waltes et al., 2009). | R1093X |
| References  1. Chen L, Trujillo KM, Van Komen S, Roh DH, Krejci L, Lewis LK, et al. Effect of amino acid substitutions in the rad50 ATP binding domain on DNA double strand break repair in yeast. J Biol Chem. 2005;280: 2620–2627. doi:10.1074/jbc.M410192200  2. De La Rosa Metzere Bierlein, and Scott W. Nelson. An Interaction between the Walker A and D-loop Motifs Is Critical to ATP Hydrolysis and Cooperativity in Bacteriophage. J Biol Chem. 2011;286: 26258–26266. doi:10.1074/jbc.M111.256305  3. Alani E, Padmore R, Kleckner N. Analysis of wild-type and rad50 mutants of yeast suggests an intimate relationship between meiotic chromosome synapsis and recombination. Cell. 1990;61: 419–436. doi:10.1016/0092-8674(90)90524-I  4. Bender CF, Sikes ML, Sullivan R, Huye LE, Le Beau MM, Roth DB, et al. Cancer predisposition and hematopoietic failure in Rad50S/S mice. Genes Dev. 2002;16: 2237–2251. doi:10.1101/gad.1007902  5. Koroleva O, Makharashvili N, Courcelle CT, Courcelle J, Korolev S. Structural conservation of RecF and Rad50: implications for DNA recognition and RecF function. EMBO J. 2007;26: 867–77. doi:10.1038/sj.emboj.7601537  6. Rojowska A, Lammens K, Seifert FU, Direnberger C, Feldmann H. Structure of the Rad 50 DNA double-strand break repair protein in complex with DNA. EMBO J. 2014;33: 2847–2859. doi:10.15252/embj.201488889  7. Roset R, Inagaki A, Hohl M, Brenet F, Lafrance-Vanasse J, Lange J, et al. The Rad50 hook domain regulates DNA damage signaling and tumorigenesis. Genes Dev. 2014;28: 451–462. doi:10.1101/gad.236745.113  8. Cahill D, Carney JP. Dimerization of the Rad50 protein is independent of the conserved hook domain. Mutagenesis. 2007;22: 269–274. doi:10.1093/mutage/gem011  9. He J, Shi LZ, Truong LN, Lu CS, Razavian N, Li Y, et al. Rad50 zinc hook is important for the Mre11 complex to bind chromosomal DNA double-stranded breaks and initiate various DNA damage responses. J Biol Chem. 2012;287: 31747–31756. doi:10.1074/jbc.M112.384750  10. Barfoot T, Herdendorf TJ, Behning BR, Stohr BA, Gao Y, Kreuzer KN, et al. Functional analysis of the bacteriophage T4 Rad50 Homolog (gp46) Coiled-coil Domain. J Biol Chem. 2015;290: 23905–23915. doi:10.1074/jbc.M115.675132  11. Hohl M, Kocha??czyk T, Tous C, Aguilera A, KrEzel A, Petrini JHJ. Interdependence of the Rad50 Hook and Globular domain functions. Mol Cell. 2015;57: 479–492. doi:10.1016/j.molcel.2014.12.018  12. Gatei M, Jakob B, Chen P, Kijas AW, Becherel OJ, Gueven N, et al. ATM protein-dependent phosphorylation of Rad50 protein Regulates DNA repair and cell cycle control. J Biol Chem. 2011;286: 31542–31556. doi:10.1074/jbc.M111.258152  13. Deshpande RA, Williams GJ, Limbo O, Williams RS, Kuhnlein J, Lee JH, et al. ATP-driven Rad50 conformations regulate DNA tethering, end resection, and ATM checkpoint signaling. EMBO J. 2014;33: 482–500. doi:10.1002/embj.201386100  14. Williams GJ, Williams RS, Williams JS, Moncalian G, Arvai AS, Limbo O, et al. ABC ATPase signature helices in Rad50 link nucleotide state to Mre11 interface for DNA repair. Nat Struct Mol Biol. 2011;18: 423–431. doi:10.1038/nsmb0911-1084c  15. Moncalian G, Lengsfeld B, Bhaskara V, Hopfner KP, Karcher A, Alden E, et al. The Rad50 Signature Motif: Essential to ATP Binding and Biological Function. J Mol Biol. 2004;335: 937–951. doi:10.1016/j.jmb.2003.11.026  16. Herdendorf TJ, Nelson SW. Functional evaluation of bacteriophage T4 Rad50 signature motif residues. Biochemistry. 2011;50: 6030–6040. doi:10.1021/bi200184w  17. Bhaskara V, Dupr A, Lengsfeld B, Hopkins BB, Chan A, Lee JH, et al. Rad50 Adenylate Kinase Activity Regulates DNA Tethering by Mre11/Rad50 Complexes. Mol Cell. 2007;25: 647–661. doi:10.1016/j.molcel.2007.01.028  18. Waltes R, Kalb R, Gatei M, Kijas AW, Stumm M, Sobeck A, et al. Human RAD50 Deficiency in a Nijmegen Breakage Syndrome-like Disorder. Am J Hum Genet. 2009;84: 605–616. doi:10.1016/j.ajhg.2009.04.010 | | | | |
