# Supplementary material for: In silico analysis on the functional and structural impact of Rad50 mutations involved in DNA strand break repair
Source: PeerJ. 2020 May 22;8:e9197. doi: 10.7717/peerj.9197 (PMC7247530; doi:10.7717/peerj.9197)
Supplement: Supplemental Information 6 — Green and red indicate neutral and deleterious effect of a mutation in Rad50, respectively. The percentage (%) shows the accuracy of each prediction using different software. [file peerj-08-9197-s006.docx]

| Motif | Mutation | Predict  SNP (%) | MAPP  (%) | PhD-  SNP (%) | Poly-Phen1  (%) | Poly-Phen2 (%) | SIFT  (%) | SNAP  (%) |
| --- | --- | --- | --- | --- | --- | --- | --- | --- |
| Walker A | P37A | 65 | 65 | 89 | 67 | 64 | 46 | 56 |
|  | N38A | 87 | 72 | 77 | 74 | 81 | 79 | 89 |
|  | G41D | 89 | 76 | 88 | 74 | 81 | 79 | 87 |
|  | K42R | 87 | 62 | 73 | 74 | 81 | 79 | 89 |
|  | K42M | 87 | 77 | 86 | 74 | 81 | 79 | 89 |
|  | K42E | 87 | 66 | 86 | 74 | 81 | 79 | 87 |
|  | K42A | 87 | 82 | 82 | 74 | 81 | 79 | 85 |
| Q-loop | Q159H | 87 | 62 | 82 | 74 | 65 | 79 | 85 |
| Zinc hook | S635A | 75 | 71 | 83 | 74 | 69 | 76 | 71 |
|  | S635G | 75 | 75 | 83 | 74 | 71 | 76 | 58 |
|  | S679R | 75 | 70 | 68 | 67 | 72 | 79 | 58 |
|  | C680G | 60 | 77 | 51 | 59 | 70 | 45 | 56 |
|  | C680N | 60 | 76 | 58 | 59 | 63 | 53 | 56 |
|  | C681G | 87 | 78 | 82 | 74 | 65 | 79 | 62 |
|  | C681A | 87 | 92 | 77 | 74 | 59 | 79 | 62 |
|  | C681S | 76 | 77 | 73 | 74 | 59 | 79 | 50 |
|  | P682R | 55 | 48 | 72 | 67 | 55 | 79 | 56 |
|  | P682E | 65 | 46 | 78 | 74 | 65 | 79 | 56 |
|  | P682A | 63 | 71 | 78 | 67 | 59 | 79 | 55 |
|  | V683R | 89 | 92 | 45 | 74 | 43 | 79 | 72 |
|  | V683I | 74 | 77 | 78 | 67 | 63 | 43 | 55 |
|  | C684G | 87 | 78 | 86 | 74 | 81 | 79 | 72 |
|  | C684A | 87 | 92 | 86 | 74 | 81 | 79 | 62 |
|  | C684R | 87 | 88 | 88 | 74 | 81 | 79 | 85 |
|  | C684S | 87 | 77 | 88 | 74 | 81 | 79 | 56 |
|  | Q685S | 74 | 74 | 83 | 67 | 79 | 53 | 67 |
|  | R686A | 76 | 62 | 51 | 74 | 59 | 79 | 56 |
| Signature motif | S1202A | 87 | 91 | 73 | 74 | 68 | 79 | 85 |
|  | S1202R | 87 | 88 | 88 | 74 | 81 | 79 | 87 |
|  | S1202M | 87 | 77 | 77 | 74 | 81 | 79 | 89 |
|  | A1203G | 87 | 77 | 77 | 59 | 81 | 79 | 56 |
|  | Q1205E | 76 | 86 | 86 | 67 | 68 | 79 | 72 |
|  | K1206M | 87 | 78 | 86 | 74 | 81 | 79 | 85 |
|  | K1206A | 87 | 82 | 86 | 74 | 81 | 79 | 81 |
|  | K1206E | 87 | 77 | 88 | 59 | 63 | 79 | 81 |
|  | K1206G | 87 | 84 | 88 | 74 | 81 | 79 | 81 |
|  | L1211W | 87 | 77 | 68 | 74 | 81 | 79 | 72 |
|  | R1214A | 87 | 84 | 88 | 74 | 68 | 79 | 56 |
|  | R1214E | 87 | 82 | 88 | 74 | 81 | 79 | 85 |
|  | R1214L | 87 | 84 | 89 | 74 | 81 | 79 | 72 |
|  | R1214W | 87 | 77 | 88 | 74 | 81 | 79 | 81 |
|  | L1215F | 76 | 63 | 61 | 74 | 81 | 79 | 55 |
| Walker B | D1231N | 87 | 77 | 88 | 74 | 81 | 53 | 81 |
|  | E1232Q | 87 | 75 | 88 | 74 | 81 | 53 | 81 |
| D-loop | D1238N | 87 | 77 | 77 | 74 | 81 | 79 | 85 |
|  | D1238A | 87 | 91 | 88 | 74 | 81 | 79 | 85 |
|  | E1240Q | 60 | 75 | 51 | 59 | 40 | 53 | 58 |
|  | N1241A | 87 | 84 | 88 | 59 | 81 | 79 | 72 |
| ATPase domain | K6E | 72 | 57 | 68 | 59 | 59 | 61 | 56 |
|  | S14P | 87 | 77 | 86 | 74 | 81 | 79 | 85 |
|  | K22M | 74 | 76 | 72 | 67 | 70 | 43 | 58 |
|  | Q23K | 63 | 70 | 51 | 67 | 50 | 67 | 72 |
|  | T65E | 72 | 86 | 66 | 59 | 40 | 79 | 62 |
|  | Q81K | 60 | 51 | 88 | 67 | 59 | 71 | 67 |
|  | R83I | 63 | 76 | 72 | 67 | 63 | 79 | 55 |
|  | S99P | 76 | 57 | 83 | 74 | 60 | 45 | 50 |
|  | V101K | 83 | 65 | 58 | 67 | 87 | 71 | 71 |
|  | Q174A | 65 | 57 | 72 | 67 | 70 | 46 | 55 |
|  | T191D | 68 | 41 | 55 | 67 | 63 | 43 | 61 |
|  | Q194S | 83 | 75 | 89 | 67 | 87 | 63 | 67 |
|  | M208C | 75 | 65 | 72 | 74 | 70 | 65 | 50 |
|  | K256P | 61 | 59 | 51 | 74 | 45 | 75 | 62 |
|  | M293A | 65 | 59 | 78 | 67 | 79 | 45 | 55 |
|  | S603Y | 63 | 65 | 78 | 59 | 61 | 79 | 50 |
|  | K921V | 60 | 68 | 68 | 59 | 40 | 53 | 55 |
|  | L673V | 63 | 68 | 83 | 67 | 50 | 79 | 67 |
|  | L694Q | 63 | 76 | 66 | 59 | 63 | 79 | 58 |
|  | V697F | 65 | 76 | 83 | 67 | 76 | 53 | 56 |
|  | Q886I | 52 | 43 | 83 | 74 | 59 | 53 | 58 |
|  | S936P | 60 | 56 | 51 | 59 | 68 | 69 | 56 |
|  | C990S | 74 | 70 | 83 | 67 | 71 | 46 | 61 |
|  | N1028P | 74 | 57 | 72 | 67 | 73 | 74 | 50 |
|  | K132E | 79 | 66 | 45 | 74 | 59 | 79 | 81 |
|  | T191E | 74 | 43 | 72 | 67 | 73 | 76 | 77 |
|  | C221E | 83 | 77 | 72 | 67 | 79 | 77 | 61 |
|  | K105E | 72 | 65 | 77 | 74 | 60 | 46 | 62 |
|  | S106E | 83 | 66 | 78 | 67 | 87 | 79 | 55 |
|  | G1199E | 87 | 88 | 88 | 74 | 81 | 79 | 81 |
|  | E110K | 74 | 71 | 68 | 67 | 64 | 43 | 58 |
|  | K126E | 60 | 57 | 58 | 67 | 79 | 76 | 56 |
|  | V127E | 55 | 65 | 77 | 67 | 41 | 53 | 72 |
|  | K122E | 74 | 65 | 78 | 67 | 79 | 76 | 56 |
|  | R1198E | 87 | 66 | 77 | 74 | 81 | 79 | 89 |
|  | Y1184R | 87 | 88 | 88 | 74 | 81 | 79 | 72 |
| SNPs | K616E | 83 | 75 | 78 | 67 | 70 | 61 | 61 |
|  | T191I | 74 | 75 | 72 | 67 | 70 | 46 | 67 |
|  | R1038G | 65 | 63 | 89 | 67 | 79 | 74 | 56 |
|  | K973M | 65 | 63 | 78 | 59 | 40 | 53 | 67 |
|  | V842A | 74 | 41 | 83 | 67 | 73 | 67 | 71 |
|  | V127I | 68 | 75 | 72 | 67 | 40 | 45 | 50 |
|  | V697A | 65 | 59 | 78 | 67 | 79 | 45 | 58 |
|  | R224H | 63 | 74 | 68 | 67 | 40 | 79 | 50 |
|  | Y964H | 55 | 76 | 59 | 74 | 55 | 45 | 55 |
|  | R193W | 65 | 43 | 78 | 74 | 68 | 79 | 81 |
|  | I94L | 83 | 74 | 78 | 67 | 87 | 71 | 58 |
|  | G469A | 83 | 70 | 83 | 67 | 61 | 71 | 67 |
|  | V315L | 74 | 80 | 78 | 67 | 71 | 46 | 71 |
