# Supplementary material for: In silico analysis on the functional and structural impact of Rad50 mutations involved in DNA strand break repair
Source: PeerJ. 2020 May 22;8:e9197. doi: 10.7717/peerj.9197 (PMC7247530; doi:10.7717/peerj.9197)
Supplement: Supplemental Information 7 — Analysis predict the effect of amino acid substitutions within Rad50 which disrupt their molecular functions. Higher score indicates higher confident level that each mutation will be deleterious to the protein functions. Data indicates most deleterious mutations happen at the conserved motif site of Rad50. [file peerj-08-9197-s007.docx]

| Motif | Mutation | Score | Affected PROSITE and ELM Motifs | |
| --- | --- | --- | --- | --- |
| Walker A | P37A | 0.536 | PS00008  PS00017 | -N-myristoylation site  -ATP/GTP-binding site motif A (P-loop) |
|  | N38A | 0.963 | PS00017 | -ATP/GTP-binding site motif A (P-loop) |
|  | G41D | 0.961 | ELME000052  ELME000064  PS00008  PS00017 | - FHA phosphopeptide ligands  - CK2 Phosphorylation site  -N-myristoylation site  -ATP/GTP-binding site motif A (P-loop) |
|  | K42R | 0.852 | ELME000052, ELME000062, ELME000064, ELME000220,  PS00008,  PS00017 | - FHA phosphopeptide ligands  - PKA Phosphorylation site  - CK2 Phosphorylation site  - FHA phosphopeptide ligands  -N-myristoylation site  -ATP/GTP-binding site motif A (P-loop)  -ATP/GTP-binding site motif A (P-loop) |
|  | K42M | 0.930 | ELME000052, ELME000064, ELME000220,  PS00008,  PS00017 | - FHA phosphopeptide ligands  - CK2 Phosphorylation site  - FHA phosphopeptide ligands  -N-myristoylation site  -ATP/GTP-binding site motif A (P-loop) |
|  | K42E | 0.939 | ELME000052, ELME000064, ELME000147, ELME000220,  PS00008,  PS00017 | - FHA phosphopeptide ligands  - CK2 Phosphorylation site  - Polo-like kinase phosphorylation site  - FHA phosphopeptide ligands  -N-myristoylation site  -ATP/GTP-binding site motif A (P-loop) |
|  | K42A | 0.956 | ELME000052  ELME000064  ELME000220  PS00008  PS00017 | - FHA phosphopeptide ligands  - CK2 Phosphorylation site  - FHA phosphopeptide ligands  -N-myristoylation site  -ATP/GTP-binding site motif A (P-loop) |
| Q-loop | Q159H | 0.466 | None | None |
| Zinc hook | S635A | 0.088 | None | None |
|  | S635G | 0.133 | None | None |
|  | S679R | 0.250 | None | None |
|  | C680G | 0.671 | ELME000070, ELME000085,  PS00001,  PS00008 | - N-glycosylation site  - Glycosaminoglycan attachment site  -N-glycosylation site  -N-myristoylation site |
|  | C680N | 0.740 | ELME000070  PS00001 | - N-glycosylation site  -N-glycosylation site |
|  | C681G | 0.760 | ELME000070 | - N-glycosylation site |
|  | C681A | 0.709 | ELME000070 | - N-glycosylation site |
|  | C681S | 0.640 | ELME000070, ELME000136, ELME000159 | - N-glycosylation site  - Proline-directed phosphorylation  - MAPK Phosphorylation Site |
|  | P682R | 0.599 | None | None |
|  | P682E | 0.536 | ELME000064  PS00006 | - CK2 Phosphorylation site  -Casein kinase II phosphorylation site |
|  | P682A | 0.385 | None | None |
|  | V683R | 0.606 | None | None |
|  | V683I | 0.127 | None | None |
|  | C684G | 0.847 | None | None |
|  | C684A | 0.798 | None | None |
|  | C684R | 0.868 | ELME000012 | di Arginine retention/retrieving signal |
|  | C684S | 0.738 | ELME000202  PS00005 | - PIKK phosphorylation site  - Protein kinase C phosphorylation site |
|  | Q685S | 0.674 | ELME000197  ELME000239 | - BRCT phosphopeptide ligands  - USP7 binding motif |
|  | R686A | 0.796 | None | None |
| Signature motif | S1202A | 0.780 | ELME000062, ELME000085 | - PKA Phosphorylation site  - Glycosaminoglycan attachment site |
|  | S1202R | 0.937 | ELME000062, ELME000085 | - PKA Phosphorylation site  - Glycosaminoglycan attachment site |
|  | S1202M | 0.927 | ELME000062, ELME000085 | - PKA Phosphorylation site  - Glycosaminoglycan attachment site |
|  | A1203G | 0.780 | ELME000062, ELME000085 | - PKA Phosphorylation site  - Glycosaminoglycan attachment site |
|  | Q1205E | 0.835 | ELME000062, ELME000064,  PS00006 | - PKA Phosphorylation site  - CK2 Phosphorylation site  - Casein kinase II phosphorylation site |
|  | K1206M | 0.833 | None | None |
|  | K1206A | 0.901 | None | None |
|  | K1206E | 0.873 | ELME000117, ELME000193 | - TRAF2 binding site  - NES Nuclear Export Signal |
|  | K1206G | 0.919 | PS00008 | N-myristoylation site |
|  | L1211W | 0.914 | ELME000335 | - SUMO interaction site |
|  | R1214A | 0.961 | PS00211 | - ATP-binding cassette, ABC transporter-type, signature and profile |
|  | R1214E | 0.956 | ELME000333,  PS00211 | - SUMO interaction site  - ATP-binding cassette, ABC transporter-type, signature and profile |
|  | R1214L | 0.952 | ELME000045, ELME000335,  PS00211 | - Nuclear receptor box  - SUMO interaction site  - ATP-binding cassette, ABC transporter-type, signature and profile |
|  | R1214W | 0.950 | PS00211 | - ATP-binding cassette, ABC transporter-type, signature and profile |
|  | L1215F | 0.889 | None | None |
| Walker B | D1231N | 0.931 | ELME000333 | - SUMO interaction site |
|  | E1232Q | 0.882 | ELME000052, ELME000333 | -FHA phosphopeptide ligands  - SUMO interaction site |
| D-loop | D1238N | 0.895 | ELME000052, ELME000220,  PS00006 | - FHA phosphopeptide ligands  -FHA phosphopeptide ligands  - Casein kinase II phosphorylation site |
|  | D1238A | 0.931 | ELME000052, ELME000220,  PS00006 | - FHA phosphopeptide ligands  - FHA phosphopeptide ligands  - Casein kinase II phosphorylation site |
|  | E1240Q | 0.492 | None | None |
|  | N1241A | 0.900 | ELME000239 | -USP7 binding motif |
| ATPase domain | K6E | 0.134 | None | None |
|  | S14P | 0.941 | ELME000235 | RRM domain ligands |
|  | K22M | 0.317 | None | None |
|  | Q23K | 0.664 | ELME000137, ELME000233 | - PP1-docking motif RVXF  - MAPK docking motifs |
|  | T65E | 0.845 | ELME000053, ELME000155,  PS00008 | - GSK3 phosphorylation site  - SH3 ligand  - N-myristoylation site |
|  | Q81K | 0.814 | ELME000137, ELME000233 | - PP1-docking motif RVXF  - MAPK docking motifs |
|  | R83I | 0.754 | ELME000137 | - PP1-docking motif RVXF |
|  | S99P | 0.739 | ELME000053, ELME000155 | - GSK3 phosphorylation site  - SH3 ligand |
|  | V101K | 0.254 | None | None |
|  | Q174A | 0.423 | None | None |
|  | T191D | 0.515 | ELME000337  PS00005 | - PP1-docking motif RVXF  - Protein kinase C phosphorylation site |
|  | Q194S | 0.420 | None | None |
|  | M208C | 0.216 | None | None |
|  | K256P | 0.215 | None | None |
|  | M293A | 0.347 | None | None |
|  | S603Y | 0.211 | None | None |
|  | K921V | 0.397 | None | None |
|  | L673V | 0.289 | None | None |
|  | L694Q | 0.579 | ELME000220, ELME000313 | - FHA phosphopeptide ligands  - Actin-binding motifs |
|  | V697F | 0.300 | None | None |
|  | Q886I | 0.639 | None | None |
|  | S936P | 0.395 | None | None |
|  | C990S | 0.209 | None | None |
|  | N1028P | 0.237 | None | None |
|  | K132E | 0.862 | ELME000063, PS00005 | - CK1 Phosphorylation site  -Protein kinase C phosphorylation site |
|  | T191E | 0.561 | ELME000117, ELME000337, PS00005 | - TRAF2 binding site  - NEK2 phosphorylation site  - Protein kinase C phosphorylation site |
|  | C221E | 0.269 | None | None |
|  | K105E | 0.430 | None | None |
|  | S106E | 0.152 | None | None |
|  | G1199E | 0.945 | ELME000012, ELME000062 | - di Arginine retention/retrieving signal  - PKA Phosphorylation site |
|  | E110K | 0.362 | None | None |
|  | K126E | 0.340 | None | None |
|  | V127E | 0.590 | ELME000233 | -MAPK docking motifs |
|  | K122E | 0.475 | None | None |
|  | R1198E | 0.944 | ELME000012 | - di Arginine retention/retrieving signal |
|  | Y1184R | 0.961 | ELME000012, ELME000102, ELME000103, ELME000108, ELME000120 | - di Arginine retention/retrieving signal  - NRD cleavage site  - PCSK cleavage site  - PCSK cleavage site  - PCSK cleavage site |
| SNPs | K616E | 0.314 | None | None |
|  | T191I | 0.387 | None | None |
|  | R1038G | 0.406 | None | None |
|  | K973M | 0.477 | None | None |
|  | V842A | 0.158 | None | None |
|  | V127I | 0.107 | None | None |
|  | V697A | 0.174 | None | None |
|  | R224H | 0.140 | None | None |
|  | Y964H | 0.436 | None | None |
|  | R193W | 0.537 | ELME000337  PS00005 | - NEK2 phosphorylation site  - Protein kinase C phosphorylation site |
|  | I94L | 0.195 | None | None |
|  | G469A | 0.326 | None | None |
|  | V315L | 0.227 | None | None |
