# Supplementary material for: In silico analysis on the functional and structural impact of Rad50 mutations involved in DNA strand break repair
Source: PeerJ. 2020 May 22;8:e9197. doi: 10.7717/peerj.9197 (PMC7247530; doi:10.7717/peerj.9197)
Supplement: Supplemental Information 8 — Red predicts the impact of mutation which cause either increase or decrease of protein or amyloid aggregation of Rad50 if mutation at the respective site occurs. [file peerj-08-9197-s008.docx]

|  |  | Protein aggregation | | Amyloid aggregation | | Hsp70 chaperone binding | |
| --- | --- | --- | --- | --- | --- | --- | --- |
| Motif | Mutation | dTANGO | Effect | dWALTZ | Effect | dLIMBO | Effect |
| Walker A | P37A | 40.42 | No | 0.02 | No | 0.00 | No |
|  | N38A | 3.70 | No | 0.00 | No | 0.00 | No |
|  | G41D | 8.06 | No | 0.00 | No | 0.00 | No |
|  | K42R | -0.01 | No | -0.04 | No | 0.00 | No |
|  | K42M | 10.38 | No | 0.15 | No | 0.00 | No |
|  | K42E | -2.73 | No | 0.09 | No | 0.00 | No |
|  | K42A | 8.30 | No | 0.83 | No | 0.00 | No |
| Q-loop | Q159H | 0.00 | No | -0.46 | No | 0.00 | No |
| Zinc hook | S635A | 0.00 | No | 0.00 | No | 0.00 | No |
|  | S635G | 0.00 | No | 0.00 | No | 0.00 | No |
|  | S679R | 0.69 | No | -0.08 | No | 0.00 | No |
|  | C680G | 0.00 | No | 0.00 | No | 0.00 | No |
|  | C680N | 0.00 | No | 0.00 | No | 0.00 | No |
|  | C681G | 0.00 | No | 0.00 | No | 0.00 | No |
|  | C681A | 0.00 | No | 0.00 | No | 0.00 | No |
|  | C681S | 0.00 | No | 0.00 | No | 0.00 | No |
|  | P682R | 0.69 | No | -0.07 | No | 0.00 | No |
|  | P682E | 0.00 | No | 0.00 | No | 0.00 | No |
|  | P682A | 0.00 | No | 0.00 | No | 0.00 | No |
|  | V683R | 0.69 | No | -0.07 | No | 0.00 | No |
|  | V683I | 0.00 | No | 0.00 | No | 0.00 | No |
|  | C684G | 0.00 | No | 0.09 | No | 0.00 | No |
|  | C684A | 0.00 | No | 0.22 | No | 0.00 | No |
|  | C684R | 0.69 | No | -0.07 | No | 0.00 | No |
|  | C684S | 0.00 | No | 0.02 | No | 0.00 | No |
|  | Q685S | 0.00 | No | 0.00 | No | 0.00 | No |
|  | R686A | 3.44 | No | 0.10 | No | 0.00 | No |
| Signature motif | S1202A | -0.01 | No | 0.00 | No | 0.00 | No |
|  | S1202R | 2.50 | No | 0.03 | No | 0.00 | No |
|  | S1202M | 0.32 | No | 0.00 | No | 0.00 | No |
|  | A1203G | 2.71 | No | 0.21 | No | 0.00 | No |
|  | Q1205E | 335.75 | Increase | -53.71 | Decrease | 0.00 | No |
|  | K1206M | 345.28 | Increase | -47.87 | Decrease | 0.00 | No |
|  | K1206A | 314.83 | Increase | -43.90 | Decrease | 0.00 | No |
|  | K1206E | 89.21 | Increase | -42.36 | Decrease | 0.00 | No |
|  | K1206G | 194.91 | Increase | -29.05 | No | 0.00 | No |
|  | L1211W | 267.65 | Increase | 167.86 | Increase | 0.00 | No |
|  | R1214A | 546.66 | Increase | -69.23 | Decrease | 0.00 | No |
|  | R1214E | 61.87 | Increase | -29.29 | No | 0.00 | No |
|  | R1214L | 550.59 | Increase | -87.31 | Decrease | 0.00 | No |
|  | R1214W | 557.4 | Increase | -91.52 | Decrease | 0.00 |  |
|  | L1215F | 245.42 | Increase | -21.64 | No | 0.00 | No |
| Walker B | D1231N | 6.74 | No | -0.04 | No | 0.00 | No |
|  | E1232Q | 4.37 | No | -0.25 | No | 00.0 | No |
| D-loop | D1238N | 0.02 | No | 0.73 | No | 0.00 | No |
|  | D1238A | 0.05 | No | 1.63 | No | 0.00 | No |
|  | E1240Q | 0.03 | No | 0.96 | No | 0.00 | No |
|  | N1241A | -0.09 | No | -3.30 | No | 0.00 | No |
| ATPase domain | K6E | 6.36 | No | 0.07 | No | 0.00 | No |
|  | S14P | 0.00 | No | 0.00 | No | 0.00 | No |
|  | K22M | 64.69 | Increase | -12.71 | No | 0.00 | No |
|  | Q23K | -161.56 | Decrease | -1.60 | No | 0.00 | No |
|  | T65E | 0.00 | No | -0.41 | No | 0.00 | No |
|  | Q81K | 0.69 | No | -0.07 | No | 0.00 | No |
|  | R83I | 182.74 | Increase | 58.48 | Increase | 0.00 | No |
|  | S99P | 0.12 | No | 0.11 | No | 0.00 | No |
|  | V101K | 0.64 | No | -0.11 | No | 0.00 | No |
|  | Q174A | 0.00 | No | 0.43 | No | 0.00 | No |
|  | T191D | -0.07 | No | -1.69 | No | 0.00 | No |
|  | Q194S | 0.02 | No | 0.21 | No | 0.00 | No |
|  | M208C | 0.00 | No | -1.85 | No | 0.00 | No |
|  | K256P | -0.69 | No | 0.17 | No | 0.00 | No |
|  | M293A | 0.00 | No | 0.01 | No | 0.00 | No |
|  | S603Y | 0.00 | No | 9.87 | No | 0.00 | No |
|  | K921V | -0.69 | No | 0.29 | No | 0.00 | No |
|  | L673V | 11.74 | No | -0.21 | No | 0.00 | No |
|  | L694Q | 0.00 | No | -5.74 | No | 0.00 | No |
|  | V697F | 0.00 | No | 239.39 | Increase | 0.00 | No |
|  | Q886I | 0.00 | No | 0.00 | No | 0.00 | No |
|  | S936P | 0.00 | No | -0.06 | No | 0.00 | No |
|  | C990S | 0.49 | No | 0.00 | No | 0.00 | No |
|  | N1028P | 0.00 | No | 0.00 | No | 0.00 | No |
|  | K132E | -0.69 | No | 0.01 | No | 0.00 | No |
|  | T191E | -0.13 | No | -2.90 | No | 0.00 | No |
|  | C221E | 0.00 | No | 0.00 | No | 0.00 | No |
|  | K105E | -0.68 | No | 0.07 | No | 0.00 | No |
|  | S106E | 0.02 | No | 0.00 | No | 0.00 | No |
|  | G1199E | -0.99 | No | 0.00 | No | 0.00 | No |
|  | E110K | -1.00 | No | -0.07 | No | 0.00 | No |
|  | K126E | -0.69 | No | 0.06 | No | 0.00 | No |
|  | V127E | 0.00 | No | -0.01 | No | 0.00 | No |
|  | K122E | -0.68 | No | 0.07 | No | 0.00 | No |
|  | R1198E | -0.69 | No | 0.07 | No | 0.00 | No |
|  | Y1184R | 0.69 | No | -0.15 | No | 0.00 | No |
| SNPs | K616E | -0.69 | No | 0.08 | No | 0.00 | No |
|  | T191I | 1.56 | No | 0.73 | No | 0.00 | No |
|  | R1038G | -0.69 | No | 0.07 | No | 0.00 | No |
|  | K973M | -0.25 | No | 0.07 | No | 0.00 | No |
|  | V842A | 0.00 | No | 0.02 | No | 0.00 | No |
|  | V127I | 0.00 | No | 0.06 | No | 0.00 | No |
|  | V697A | 0.00 | No | 55.56 | Increase | 0.00 | No |
|  | R224H | -0.61 | No | 0.28 | No | 0.00 | No |
|  | Y964H | 0.08 | No | -111.51 | Decrease | 0.00 | No |
|  | R193W | 7.11 | No | 0.71 | No | 0.00 | No |
|  | I94L | -9.52 | No | -18.37 | No | 0.00 | No |
|  | G469A | 0.00 | No | 0.29 | No | 0.00 | No |
|  | V315L | 0.00 | No | 0.00 | No | 0.00 | No |
