# Supplementary material for: In silico analysis on the functional and structural impact of Rad50 mutations involved in DNA strand break repair
Source: PeerJ. 2020 May 22;8:e9197. doi: 10.7717/peerj.9197 (PMC7247530; doi:10.7717/peerj.9197)
Supplement: Supplemental Information 9 — Most mutations at the motif of Rad50 were predicted to cause protein instability. [file peerj-08-9197-s009.docx]

|  |  | I-Mutant | | MuPro | |
| --- | --- | --- | --- | --- | --- |
| Motif | Mutation | ddG | Stability | ddG | Stability |
| Walker A | P37A | -1.60 | DECREASE | -1.16 | DECREASE |
|  | N38A | -0.41 | DECREASE | -0.30 | DECREASE |
|  | G41D | -1.08 | DECREASE | -1.27 | DECREASE |
|  | K42R | -0.24 | DECREASE | -0.11 | DECREASE |
|  | K42M | 0.00 | INCREASE | 0.07 | INCREASE |
|  | K42E | -0.37 | DECREASE | -0.05 | DECREASE |
|  | K42A | -0.61 | DECREASE | -0.29 | DECREASE |
| Q-loop | Q159H | -0.99 | DECREASE | -0.72 | DECREASE |
| Zinc hook | S635A | -0.72 | DECREASE | -0.76 | DECREASE |
|  | S635G | -1.12 | DECREASE | -1.08 | DECREASE |
|  | S679R | -0.01 | DECREASE | -0.57 | DECREASE |
|  | C680G | -1.18 | DECREASE | -1.48 | DECREASE |
|  | C680N | -0.57 | DECREASE | -1.03 | DECREASE |
|  | C681G | -1.18 | DECREASE | -1.97 | DECREASE |
|  | C681A | -0.72 | DECREASE | -1.16 | DECREASE |
|  | C681S | 0.70 | INCREASE | -1.71 | DECREASE |
|  | P682R | -0.81 | DECREASE | -0.56 | DECREASE |
|  | P682E | -0.84 | DECREASE | -0.41 | DECREASE |
|  | P682A | -1.37 | DECREASE | -0.83 | DECREASE |
|  | V683R | -0.50 | DECREASE | -0.42 | DECREASE |
|  | V683I | 0.17 | INCREASE | 0.32 | INCREASE |
|  | C684G | -1.24 | DECREASE | -1.65 | DECREASE |
|  | C684A | -0.63 | DECREASE | -1.02 | DECREASE |
|  | C684R | -0.19 | DECREASE | -0.96 | DECREASE |
|  | C684S | -0.71 | DECREASE | -1.19 | DECREASE |
|  | Q685S | -0.40 | DECREASE | -0.96 | DECREASE |
|  | R686A | -0.47 | DECREASE | -0.50 | DECREASE |
| Signature motif | S1202A | -0.45 | DECREASE | -0.28 | DECREASE |
|  | S1202R | 0.03 | INCREASE | -0.21 | INCREASE |
|  | S1202M | 0.27 | INCREASE | 0.23 | DECREASE |
|  | A1203G | -1.36 | DECREASE | -1.12 | DECREASE |
|  | Q1205E | -0.45 | DECREASE | -0.69 | DECREASE |
|  | K1206M | 0.10 | INCREASE | 0.86 | INCREASE |
|  | K1206A | -0.55 | DECREASE | 0.62 | INCREASE |
|  | K1206E | -0.22 | DECREASE | 0.45 | INCREASE |
|  | K1206G | -0.94 | DECREASE | -0.56 | DECREASE |
|  | L1211W | -1.27 | DECREASE | -1.36 | DECREASE |
|  | R1214A | -0.48 | DECREASE | -1.00 | DECREASE |
|  | R1214E | -0.29 | DECREASE | -0.80 | DECREASE |
|  | R1214L | -0.24 | DECREASE | -0.24 | DECREASE |
|  | R1214W | -0.15 | DECREASE | -0.99 | DECREASE |
|  | L1215F | -1.04 | DECREASE | -1.33 | DECREASE |
| Walker B | D1231N | -0.57 | DECREASE | -0.69 | DECREASE |
|  | E1232Q | -0.61 | DECREASE | -0.84 | DECREASE |
| D-loop | D1238N | -0.47 | DECREASE | -1.00 | DECREASE |
|  | D1238A | -0.11 | DECREASE | -1.01 | DECREASE |
|  | E1240Q | -0.58 | DECREASE | -1.40 | DECREASE |
|  | N1241A | -0.45 | DECREASE | -1.48 | DECREASE |
| ATPase domain | K6E | -0.63 | DECREASE | -0.53 | DECREASE |
|  | S14P | -0.39 | DECREASE | -1.39 | DECREASE |
|  | K22M | -0.04 | DECREASE | 0.31 | INCREASE |
|  | Q23K | -0.28 | DECREASE | -1.09 | DECREASE |
|  | T65E | -0.55 | DECREASE | -1.67 | DECREASE |
|  | Q81K | -0.65 | DECREASE | -1.38 | DECREASE |
|  | R83I | -0.02 | DECREASE | 0.26 | INCREASE |
|  | S99P | -0.15 | DECREASE | -1.13 | DECREASE |
|  | V101K | -1.16 | DECREASE | -1.38 | DECREASE |
|  | Q174A | -0.14 | DECREASE | -1.26 | DECREASE |
|  | T191D | -0.63 | DECREASE | -0.30 | DECREASE |
|  | Q194S | -0.58 | DECREASE | -0.92 | DECREASE |
|  | M208C | -0.63 | DECREASE | -1.17 | DECREASE |
|  | K256P | -0.28 | DECREASE | -1.08 | DECREASE |
|  | M293A | -1.17 | DECREASE | -1.47 | DECREASE |
|  | S603Y | -0.02 | DECREASE | -0.33 | DECREASE |
|  | K921V | 0.29 | INCREASE | 0.21 | INCREASE |
|  | L673V | -1.56 | DECREASE | -1.03 | DECREASE |
|  | L694Q | -1.64 | DECREASE | -1.51 | DECREASE |
|  | V697F | -0.92 | DECREASE | -1.31 | DECREASE |
|  | Q886I | 0.56 | INCREASE | -0.13 | DECREASE |
|  | S936P | -0.30 | DECREASE | -1.45 | DECREASE |
|  | C990S | -0.68 | DECREASE | -1.84 | DECREASE |
|  | N1028P | 0.02 | INCREASE | -1.20 | DECREASE |
|  | K132E | -0.63 | DECREASE | -0.74 | DECREASE |
|  | T191E | -0.46 | DECREASE | -0.06 | DECREASE |
|  | C221E | -0.35 | DECREASE | -0.82 | DECREASE |
|  | K105E | -0.04 | DECREASE | -0.28 | DECREASE |
|  | S106E | 0.11 | INCREASE | -0.68 | DECREASE |
|  | G1199E | -0.53 | DECREASE | -0.72 | DECREASE |
|  | E110K | -0.32 | DECREASE | -1.29 | DECREASE |
|  | K126E | -0.36 | DECREASE | 0.11 | INCREASE |
|  | V127E | -1.15 | DECREASE | -1.06 | DECREASE |
|  | K122E | -0.20 | DECREASE | -0.63 | DECREASE |
|  | R1198E | -0.47 | DECREASE | -0.87 | DECREASE |
|  | Y1184R | -1.16 | DECREASE | -1.98 | DECREASE |
| SNPs | K616E | -0.73 | DECREASE | -0.52 | DECREASE |
|  | T191I | -0.08 | DECREASE | 0.34 | DECREASE |
|  | R1038G | -0.88 | DECREASE | -2.23 | DECREASE |
|  | K973M | 0.20 | INCREASE | -0.25 | DECREASE |
|  | V842A | -1.03 | DECREASE | -1.78 | DECREASE |
|  | V127I | -0.23 | DECREASE | -0.42 | DECREASE |
|  | V697A | -0.95 | DECREASE | -2.08 | DECREASE |
|  | R224H | -0.80 | DECREASE | -1.47 | DECREASE |
|  | Y964H | -1.05 | DECREASE | -1.76 | DECREASE |
|  | R193W | 0.03 | INCREASE | -0.67 | DECREASE |
|  | I94L | -0.75 | DECREASE | -0.35 | DECREASE |
|  | G469A | -0.60 | DECREASE | -0.72 | DECREASE |
|  | V315L | -1.10 | DECREASE | -0.29 | DECREASE |
