# Supplementary material for: In silico analysis on the functional and structural impact of Rad50 mutations involved in DNA strand break repair
Source: PeerJ. 2020 May 22;8:e9197. doi: 10.7717/peerj.9197 (PMC7247530; doi:10.7717/peerj.9197)
Supplement: Supplemental Information 10 — Table indicate the predictive results of the impact of the other 51 mutation that are not considered deleterious but reported to occur in Rad50. Most bioinformatic tools used were not able to confidently predict the effect of these mutations, which located mostly at the non-conserved regions of Rad50. [file peerj-08-9197-s010.docx]

|  |  | Amino acid Impact (Neutral/Deleterious) | | | | | | | | Molecular mechanisms | | | | Biological impact | | Protein stability | |
| --- | --- | --- | --- | --- | --- | --- | --- | --- | --- | --- | --- | --- | --- | --- | --- | --- | --- |
| **Motif** | **Mutation** | **PS** | | **MP** | **Ph-S** | **PP-1** | **PP-2** | **SF** | **SP** | **MutPred2** | **Pr** | **P-value** | **Affected functional sites** | **AG** | **AM** | **IM** | **MP** |
| Walker A | P37A [2] | N | | N | N | N | N | D | D | Altered ordered interface  Loss of relative solvent accessibility  Gain of allosteric site at N38  Altered DNA binding  Altered metal binding  Gain of catalytic site at G39  Gain of methylation at K42 | 0.34  0.32  0.28  0.25  0.24  0.19  0.17 | 7.3e-03  5.8e-03  3.3e-03  8.1e-03  0.02  0.01  8.9e-03 | -N-myristoylation site  -ATP/GTP-binding site motif A (P-loop) | No | No | ↓ | ↓ |
| Zinc hook | C680G [8] | N | | N | N | D | N | D | D | Gain of N-linked glycosylation at N677 | 0.02 | 0.03 | - N-glycosylation site  - Glycosaminoglycan attachment site  -N-myristoylation site | No | No | ↓ | ↓ |
|  | P682R  [11] | D | | D | N | N | D | D | D | Gain of helix  Altered coiled coil  Loss of N-linked glycosylation at N677 | 0.32  0.09  0.02 | 2.8e-03  0.04  0.04 | None | No | No | ↓ | ↓ |
|  | C684G [8] | D | | D | D | D | D | D | D | No effect | - | - | None | No | No | ↓ | ↓ |
|  | R686A [8] | D | | D | N | D | D | D | D | No effect | - | - | None | No | No | ↓ | ↓ |
| Signature motif | A1203G [16] | D | | D | D | D | D | D | D | Gain of allosteric site at R1200  Gain of catalytic site at R1200  Gain of methylation at K1206 | 0.22  0.20  0.10 | 0.02  0.01  0.04 | - PKA Phosphorylation site  - Glycosaminoglycan attachment site | No | No | ↓ | ↓ |
|  | K1206G [13] | D | | D | D | D | D | D | D | Loss of catalytic site at K1206 | 0.09 | 0.05 | N-myristoylation site | ↑ | No | ↓ | ↓ |
|  | R1214L | D | | D | D | D | D | D | D | Loss of allosteric site at R1214 | 0.22 | 0.03 | - Nuclear receptor box  - SUMO interaction site  - ATP-binding cassette, ABC transporter-type, signature and profile | ↑ | ↓ | ↓ | ↓ |
|  | R1214W [13] | D | | D | D | D | D | D | D | Loss of allosteric site at R1214 | 0.21 | 0.04 | - ATP-binding cassette, ABC transporter-type, signature and profile | ↑ | ↓ | ↓ | ↓ |
|  | L1215F [13] | D | | D | D | D | D | D | N | Gain of allosteric site at R1214 | 0.19 | 0.05 | None | ↑ | No | ↓ | ↓ |
| Walker B | D1231N [5] | D | | D | D | D | D | D | D | Altered metal binding  Altered ordered interface  Loss of catalytic site at D1231  Loss of allosteric site at P1233  Altered transmembrane protein | 0.42  0.29  0.29  0.25  0.12 | 5.8e-03  0.03  2.3e-03  0.01  0.03 | - SUMO interaction site | No | No | ↓ | ↓ |
| D-loop  ATPase domain/ coiled-coil | E1240Q [2] | N | | N | N | D | D | D | N | No effect | - | - | None | No | No | ↓ | ↓ |
|  | N1241A  [2] | D | | D | D | D | D | D | D | Altered ordered interface  Altered metal binding  Loss of allosteric site at N1236  Altered coiled coil  Loss of catalytic site at D1238 | 0.36  0.27  0.20  0.13  0.13 | 3.8e-03  7.8e-03  0.04  0.03  0.03 | -USP7 binding motif | No | No | ↓ | ↓ |
|  | S14P [3] | D | | D | D | D | D | D | D | Altered DNA binding  Loss of allosteric site at R13  Loss of strand  Loss of proteolytic cleavage at R13 | 0.32  0.31  0.26  0.15 | 1.7e-03  3.1e-03  0.04  0.01 | RRM domain ligands | No | No | ↓ | ↓ |
|  | Q23K [3] | N | | N | N | N | D | N | D | Loss of strand  Gain of B-factor | 0.27  0.25 | 0.02  0.03 | - PP1-docking motif RVXF  - MAPK docking motifs | ↓ | No | ↓ | ↓ |
|  | T65E [3] | D | | D | N | D | D | D | D | Altered metal binding  Altered ordered interface  Loss of methylation at K62 | 0.30  0.28  0.09 | 5.5e-03  0.04  0.05 | - GSK3 phosphorylation site  - SH3 ligand  - N-myristoylation site | No | No | ↓ | ↓ |
|  | Q81K [3] | N | | D | D | N | D | N | N | Altered DNA binding  Altered stability | 0.19  0.12 | 0.02  0.03 | - PP1-docking motif RVXF  - MAPK docking motifs | No | No | ↓ | ↓ |
|  | S99P [3] | D | | D | D | D | D | D | N | Altered DNA binding  Altered ordered interface  Altered coiled coil  Altered stability | 0.37  0.30  0.11  0.11 | 6.0e-04  0.02  0.04  0.03 | - GSK3 phosphorylation site  - SH3 ligand | No | No | ↓ | ↓ |
|  | V101K [3] | N | | N | N | N | N | N | N | No effect | - | - | None | No | No | ↓ | ↓ |
|  | Q174A [13] | N | | D | N | N | N | D | N | No effect | - | - | None | No | No | ↓ | ↓ |
|  | T191D [6] | N | | D | N | N | N | D | N | Altered coiled coil  Loss of acetylation at K187  Gain of ubiquitylation at K187 | 0.29  0.28  0.15 | 0.02  5.8e-03  0.04 | - PP1-docking motif RVXF  - Protein kinase C phosphorylation site | No | No | ↓ | ↓ |
|  | Q194S [11] | N | | N | N | N | N | N | N | No effect | - | - | None | No | No | ↓ | ↓ |
|  | M208C [10] | N | | N | N | D | N | N | N | No effect | - | - | None | No | No | ↓ | ↓ |
|  | K256P [10] | D | | D | N | D | D | N | D | No effect | - | - | None | No | No | ↓ | ↓ |
|  | M293A [11] | N | | D | N | N | N | D | N | No effect | - | - | None | No | No | ↓ | ↓ |
|  | S603Y [11] | N | | N | N | D | N | D | N | No effect | - | - | None | No | No | ↓ | ↓ |
|  | K921V [3] | N | | N | N | D | D | D | N | No effect | - | - | None | No | No | ↑ | ↑ |
|  | L673V [11] | N | | N | N | N | D | D | N | No effect | - | - | None | No | No | ↓ | ↑ |
|  | L694Q [11] | N | | N | N | D | N | D | N | Gain of intrinsic disorder | 0.40 | 7.9e-03 | - FHA phosphopeptide ligands  - Actin-binding motifs | No | No | ↓ | ↓ |
|  | V697F [11] | N | | N | N | N | N | D | D | No effect | - | - | None | No | ↑ | ↓ | ↓ |
|  | Q886I [11] | D | | D | N | D | D | D | N | No effect | - | - | None | No | No | ↑ | ↓ |
|  | S936P [3] | N | | D | N | D | N | N | D | No effect | - | - | None | No | No | ↓ | ↓ |
|  | C990S [10] | N | | N | N | N | N | D | N | No effect | - | - | None | No | No | ↓ | ↓ |
|  | N1028P [10] | N | | D | N | N | N | N | N | No effect | - | - | None | No | No | ↑ | ↓ |
|  | E110K [6] | N | | N | N | N | N | D | N | No effect | - | - | None | No | No | ↓ | ↓ |
|  | K126E [6] | N | | D | N | N | N | D | D | No effect | - | - | None | No | No | ↓ | ↑ |
|  | V127E [6] | D | | N | D | N | D | D | D | Gain of intrinsic disorder  Loss of strand  Gain of B-factor  Altered transmembrane protein  Altered stability | 0.43  0.30  0.28  0.26  0.10 | 5.3e-03  2.4e-03  5.9e-03  1.3e-03  0.04 | -MAPK docking motifs |  |  | ↓ | ↓ |
|  | K122E [6] | N | | N | N | N | N | N | D | No effect | - | - | None | No | No | ↓ | ↓ |
| SNPs | I94L | N | | N | N | N | N | N | N | No effect | - | - | None | No | No | ↓ | ↓ |
|  | V127I | N | | N | N | N | D | D | N | No effect | - | - | None | No | No | ↓ | ↓ |
|  | T191I | N | | N | N | N | N | D | N | No effect | - | - | None | No | No | ↓ | ↓ |
|  | R193W | D | | D | N | D | D | D | D | Altered coiled coil | 0.93 | 6.7e-04 | - NEK2 phosphorylation site  - Protein kinase C phosphorylation site | No | No | ↑ | ↓ |
|  | R224H | N | | N | N | N | D | D | N | No effect | - | - | None | No | No | ↓ | ↓ |
|  | V315L | N | | N | N | N | N | D | N | No effect | - | - | None | No | No | ↓ | ↓ |
|  | G469A | N | | N | N | N | N | N | N | No effect | - | - | None | No | No | ↓ | ↓ |
|  | K616E | N | | N | N | N | N | N | N | No effect | - | - | None | No | No | ↓ | ↓ |
|  | V697A | N | | D | N | N | N | D | N | No effect | - | - | None | No | ↑ | ↓ | ↓ |
|  | V842A | N | | D | N | N | N | N | N | No effect | - | - | None | No | No | ↑ | ↓ |
|  | Y964H | D | | N | D | D | D | D | N | No effect | - | - | None | No | ↓ | ↓ | ↓ |
|  | K973M | N | | N | N | D | D | N | N | No effect | - | - | None | No | No | ↑ | ↓ |
|  | R1038G | N | | D | N | N | N | N | D | No effect | - | - | None | No | No | ↓ | ↓ |
| Abbreviation: | | | | | | | | | | | | | | | | | |
| IM- Imutant  MP- MuPro  PS-PredictSNP  MP-MAPP  PhS-PhD-SNP  PP1-Poly-Phen1  PP2-Poly-Phen2 | | | SF- SIFT  SN- SNAP  Pr-Probability  AG- protein aggregation  AM- Amyloid aggregation  IM- Imutant  MP-MuPro | | | | | | | | | | | | | | |
